# Nondestructive Testing of Externally Bonded FRP Concrete Structures: A Comprehensive Review

**DOI:** 10.3390/polym17091284

**Published:** 2025-05-07

**Authors:** Eyad Alsuhaibani

**Affiliations:** Department of Civil Engineering, College of Engineering, Qassim University, Buraidah 52571, Saudi Arabia; e.alsuhaibani@qu.edu.sa

**Keywords:** fiber-reinforced polymer (FRP), structural health monitoring (SHM), nondestructive testing (NDT), external strengthening, retrofitting, cost-effective inspection

## Abstract

The growing application of Fiber-Reinforced Polymer (FRP) composites in rehabilitating deteriorating concrete infrastructure underscores the need for reliable, cost-effective, and automated nondestructive testing (NDT) methods. This review provides a comprehensive analysis of existing and emerging NDT techniques used to assess externally bonded FRP (EB-FRP) systems, emphasizing their accuracy, limitations, and practicality. Various NDT methods, including Ground-Penetrating Radar (GPR), Phased Array Ultrasonic Testing (PAUT), Infrared Thermography (IRT), Acoustic Emission (AE), and Impact–Echo (IE), are critically evaluated in terms of their effectiveness in detecting debonding, voids, delaminations, and other defects. Recent technological advancements, particularly the integration of artificial intelligence (AI) and machine learning (ML) in NDT applications, have significantly improved defect characterization, automated inspections, and real-time data analysis. This review highlights AI-driven NDT approaches such as automated crack detection, hybrid NDT frameworks, and drone-assisted thermographic inspections, which enhance accuracy and efficiency in large-scale infrastructure assessments. Additionally, economic considerations and cost–performance trade-offs are analyzed, addressing the feasibility of different NDT methods in real-world FRP-strengthened structures. Finally, the review identifies key research gaps, including the need for standardization in FRP-NDT applications, AI-enhanced defect quantification, and hybrid inspection techniques. By consolidating state-of-the-art research and emerging innovations, this paper serves as a valuable resource for engineers, researchers, and practitioners involved in the assessment, monitoring, and maintenance of FRP-strengthened concrete structures.

## 1. Introduction

The global challenge of deteriorating concrete infrastructure demands innovative solutions to ensure safety, durability, and sustainability. Aging concrete structures are increasingly subjected to environmental stressors, material degradation, and operational loads, leading to a decline in their structural integrity and performance. In response to these challenges, Fiber-Reinforced Polymer (FRP) composites have emerged as a transformative solution for structural rehabilitation and strengthening. FRP materials offer a high strength-to-weight ratio, corrosion resistance, and ease of application, making them ideal for retrofitting bridges, buildings, and other structural elements [1,2,3,4,5]. The implementation of externally bonded FRP (EB-FRP) systems has significantly influenced structural rehabilitation practices, particularly in transportation and infrastructure projects. However, the effectiveness of these systems heavily depends on the quality of installation, bonding integrity, and long-term durability. Despite their advantages, FRP retrofits can develop critical defects, such as debonding, delamination, void formation, and matrix cracks, which compromise their structural efficiency. Consequently, reliable and cost-effective nondestructive testing (NDT) methods are essential to assess FRP–concrete systems without causing damage [6,7,8].

NDT plays a critical role in evaluating EB-FRP concrete structures, providing non-invasive assessment techniques for detecting defects, bond failures, and subsurface anomalies. Various NDT techniques have been developed to address these challenges, including Visual Testing (VT), Tap Testing (TT), Impact–Echo (IE), Acoustic Emission (AE), Acoustic Impact Testing (AIT), Ground-Penetrating Radar (GPR), Phased Array Ultrasonic Testing (PAUT), Ultrasonic Testing (UT), Infrared Thermography (IRT), Eddy Current Testing (ECT), and Radiographic Testing (RT). Each method offers unique advantages, from surface-level inspection to deep-penetration analysis, helping engineers and researchers assess FRP material properties, adhesion quality, and internal damage more effectively [9,10,11,12,13,14,15,16]. However, challenges such as limited penetration depth, operator dependency, and economic constraints necessitate the development of integrated and automated NDT systems. Recent advancements in structural health monitoring (SHM) have introduced real-time sensing technologies and artificial intelligence (AI)-driven inspection frameworks, revolutionizing the way FRP-strengthened structures are evaluated. AI-based techniques, including machine learning (ML) algorithms, deep learning models, and artificial neural networks (ANNs), enable automated defect classification, predictive maintenance, and enhanced data interpretation [17]. Additionally, drones equipped with AI-powered imaging are improving large-scale inspections by providing real-time analysis and defect identification, particularly in bridge monitoring and high-rise structures [18,19].

This paper provides a comprehensive analysis of current NDT techniques used in the assessment of EB-FRP concrete structures, with a strong focus on their role within SHM frameworks. The key objectives of this review are:

- Examining the capabilities and limitations of different NDT methods in detecting common EB-FRP concrete defects, including debonding, delamination, voids, and cracks.
- Evaluating the effectiveness of traditional and emerging NDT technologies, particularly in automated and hybrid inspection methods.
- Exploring practical applications, cost–performance trade-offs, and economic feasibility of NDT in real-world EB-FRP concrete structures.
- Investigating the integration of AI, ML, and automated SHM solutions to enhance defect detection, predictive maintenance, and long-term structural monitoring.

By synthesizing recent research findings and emerging innovations, this review contributes to the growing body of knowledge in SHM and NDT, aiming to serve as a valuable resource for researchers, engineers, and practitioners involved in the design, implementation, and maintenance of FRP-strengthened concrete structures.

## 2. Defects in EB-FRP Structures

**Figure 1:**
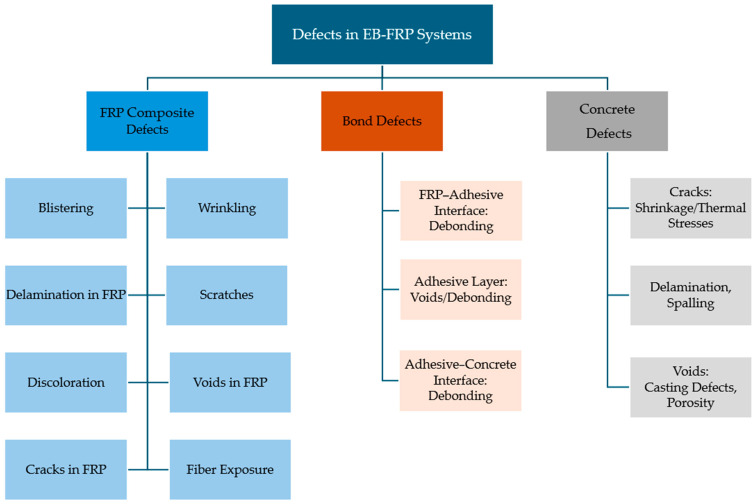
Classification of defects in EB-FRP systems.

**Figure 2:**
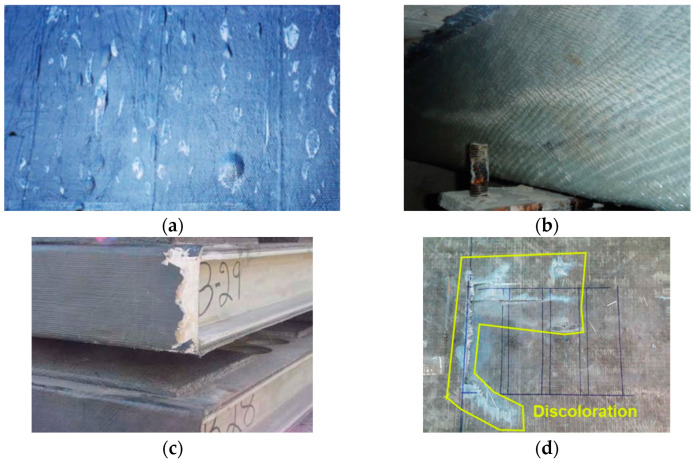

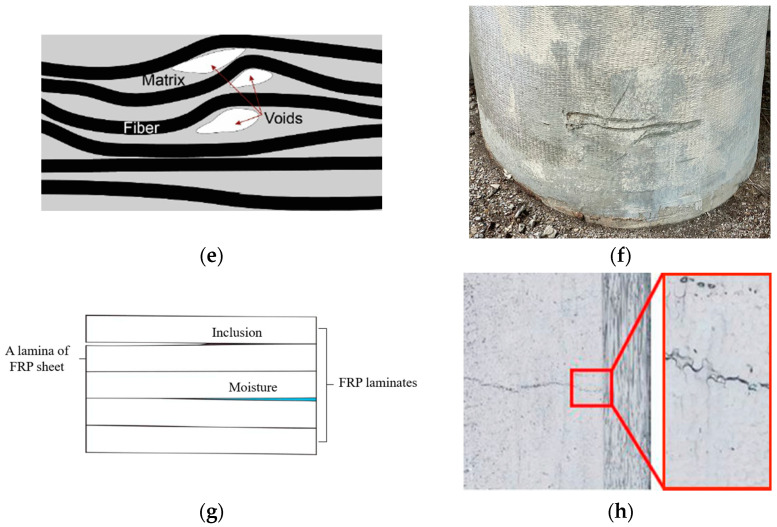
Common FRP composite defects in EB-FRP systems. (**a**) Blistering, (**b**) wrinkling, (**c**) fiber exposure from improper handling, (**d**) cracks and discoloration, (**e**) voids inside composite material at the interlaminar interfaces, (**f**) scratch caused at the externally applied FRP, (**g**) delamination in FRP laminate, (**h**) crack formation in FRP composite. All images adopted from [24].

**Figure 3:**
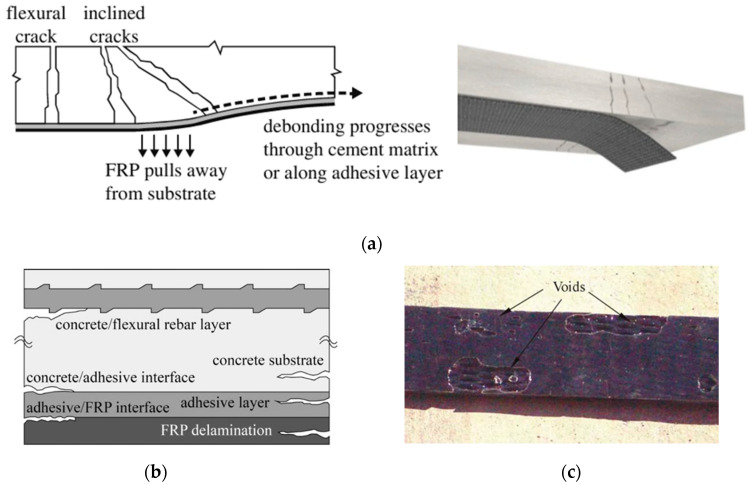
Common bond defects in EB-FRP systems. (**a**) Debonding of EB-FRP, adopted from [20,24], (**b**) paths of debonding propagation [21], (**c**) typical voids at the concrete/FRP interface [25].

**Figure 4:**
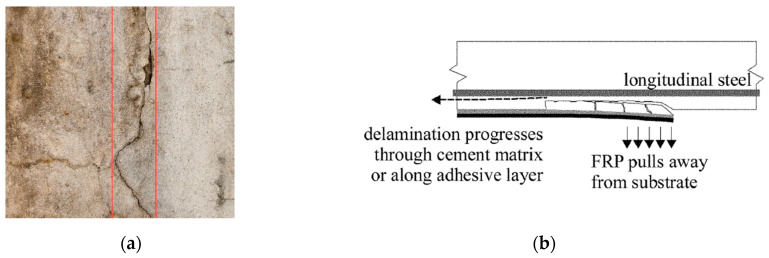
Common concrete defects in EB-FRP systems. (**a**) Concrete crack highlighted with red lines for visual emphasis [26], (**b**) concrete cover delamination [20].

The performance of EB-FRP systems in retrofitting concrete structures is strongly influenced by their design, installation quality, and interaction with the substrate. Despite their numerous advantages, such as high strength-to-weight ratios, corrosion resistance, and ease of application, EB-FRP systems are susceptible to various defects. These defects can significantly compromise structural integrity, reduce service life, and hinder their overall effectiveness. To ensure the long-term performance of these systems, understanding the types, causes, and consequences of these defects is crucial for effective maintenance and application of NDT methods. Defects in EB-FRP systems can be categorized into three primary groups: FRP composite defects, bond defects, and concrete substrate defects, as illustrated in the accompanying flowchart (Figure 1). Additionally, Figure 2, Figure 3 and Figure 4 showcase examples of typical defects for each primary group, providing a visual representation of real-world issues that occur in EB-FRP systems. Each category represents distinct challenges that can arise from fabrication errors, poor installation practices, environmental conditions, or pre-existing substrate deficiencies.

FRP composite defects are issues within the FRP material itself, which can occur either on the surface or internally. Surface defects include blistering, wrinkling, scratches, and discoloration. Blisters, caused by trapped air or moisture during installation, reduce the effective bonding area and compromise load transfer between the FRP and substrate. Wrinkling, which arises from improper tension or alignment during application, creates stress concentrations that weaken the FRP under load. Scratches or fiber exposure, often resulting from improper handling or installation, expose the composite to environmental degradation, while discoloration caused by ultraviolet radiation or chemical reactions signals potential material weakening. Internally, defects such as voids, delamination, and cracks can compromise the structural integrity of the FRP. Voids, which are air pockets formed during fabrication or curing, reduce shear and tensile strength, while delamination, caused by improper resin distribution or trapped air, leads to localized failure under stress. Cracks, often initiated by overloading or impact damage, propagate through the material, increasing the risk of sudden brittle failure. Figure 2 illustrates examples of these defects, highlighting their impact on FRP performance.

Bond defects occur at the critical interfaces between the FRP, adhesive, and concrete substrate. The FRP-adhesive interface can experience debonding due to poor surface preparation, contamination, or insufficient curing, leading to reduced load transfer efficiency. In the adhesive layer itself, voids caused by irregular resin application or curing result in weak spots that compromise the bond. Similarly, the adhesive–concrete interface is prone to debonding due to contaminants, moisture ingress, or inadequate substrate preparation. These bond defects are particularly significant as they directly affect the structural performance and durability of the retrofitted system. Examples of bond defects are depicted in Figure 3, emphasizing their role in limiting the effectiveness of EB-FRP systems. Research indicates that debonding is among the most frequently observed defects, often caused by improper surface preparation or environmental factors such as moisture exposure and thermal cycling. Studies show that premature failures in EB-FRP-retrofitted structures are due to bonding/interface defects, emphasizing the need for robust detection techniques [4,5,20,21].

Concrete substrate defects represent issues within the concrete layer that serves as the foundation for the FRP system. Cracks, whether caused by shrinkage, thermal stresses, or mechanical loading, weaken the bond and allow moisture ingress, accelerating degradation. Micro-cracking in the concrete substrate facilitates water penetration, leading to corrosion of embedded steel reinforcement, ultimately reducing the overall durability of the structure [22,23]. Voids, often due to improper casting or compaction, reduce the substrate’s structural capacity and compromise the effectiveness of the FRP bond. Additionally, delamination and spalling, caused by stress-induced separation of concrete layers or surface flaking, reduce the bond area and the overall capacity of the retrofitted element. Figure 4 provides visual examples of concrete substrate defects, illustrating their impact on the overall performance of the system.

## 3. Nondestructive Testing (NDT) Methods

### 3.1. Visual Testing (VT)

Visual Testing (VT) is among the most versatile and fundamental methods in NDT for FRP-strengthened concrete structures. As a foundational step in inspection processes, VT leverages manual or automated observations to identify visible defects, including cracks, spalling, discoloration, delamination, excessive fiber waviness, and sagging [27,28]. Its simplicity and cost-effectiveness make it indispensable, as many surface defects can be detected without requiring advanced techniques. However, VT’s reliance on human perception poses limitations, particularly in identifying subsurface anomalies. To enhance its precision and applicability, auxiliary tools such as magnifying lenses, calipers, and remote imaging devices are commonly employed, providing greater accuracy and reliability. Advanced technologies, including drones, fiber-optic cameras, and automated systems, have expanded VT’s reach, enabling inspection in hard-to-access or hazardous areas while delivering real-time data. For example, remote sensing systems, utilizing fiber-optic bundles or video cameras, facilitate the examination of enclosed or obstructed regions. High-speed cameras coupled with motion magnification techniques can detect interfacial defects in FRP-bonded concrete, offering a non-contact and efficient alternative for detailed inspections [29].

Modern innovations have further revolutionized VT, particularly in large-scale or complex structures. Drones equipped with AI-powered visual analysis and augmented reality (AR) tools significantly improve efficiency and accuracy, allowing for seamless defect detection even in challenging environments [30]. Deep learning approaches show exceptional promise in identifying and classifying cracks in concrete structures with high precision, minimizing human error and increasing consistency [31]. Furthermore, the integration of IRT with VT enables enhanced characterization of surface and near-surface defects, providing a complementary layer of diagnostic insight [32]. VT’s versatility extends beyond defect detection; it plays a critical role in monitoring adhesive bond quality and surface conditions, which are essential for ensuring long-term structural safety and performance. Studies have demonstrated that periodic VT, integrated with other NDT methods, offers a comprehensive approach to evaluating both surface and subsurface degradation [9,11,33,34,35]. Field applications underline VT’s relevance in structural maintenance and retrofitting. From bridges to urban infrastructure, VT serves as an essential tool in both routine inspections and targeted evaluations. By combining simplicity with cutting-edge advancements, VT bridges traditional inspection methods and modern technological innovations, cementing its role as a vital component of NDT for FRP-strengthened concrete structures.

### 3.2. Tap Testing (TT)

**Figure 5:**
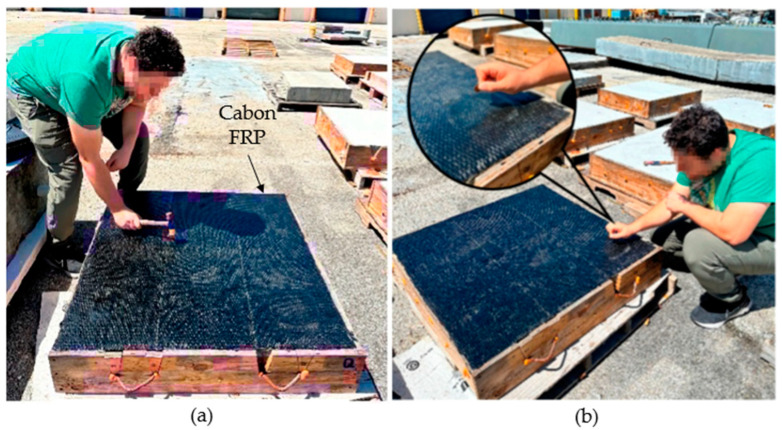
Tap testing on carbon FRP-retrofitted concrete slab. (**a**) Tapping with a hammer to detect subsurface defects, (**b**) coin tapping used to identify tonal variations indicating possible debonding. All images were adopted from [11].

Tap Testing (TT) is a simple, effective NDT method for FRP-strengthened concrete structures, used to identify defects like delamination or voids. The process involves lightly tapping the surface of the FRP-strengthened concrete with a small hammer or a similar tool and listening to the resulting sound, as illustrated in Figure 5. A clear, consistent tone typically indicates sound material, while dull or hollow sounds suggest the presence of delamination or voids. This technique relies on the ability of the inspector to differentiate tonal variations, making it a qualitative method heavily influenced by human perception. Tapping tests are advantageous due to their low cost, simplicity, and ability to provide immediate results. They are particularly useful for small-scale inspections or as a preliminary assessment tool before employing more sophisticated techniques [27]. Despite its strengths, TT is inherently qualitative, relying heavily on the inspector’s ability to differentiate tonal variations. This reliance on human perception introduces variability, making the method operator dependent. Additionally, TT cannot quantify the size, depth, or exact nature of detected defects, limiting its utility for comprehensive structural assessments [36]. To mitigate these limitations, TT is frequently combined with other NDT methods, such as ultrasonic testing and IRT, which provide detailed subsurface insights [37].

The application of tapping tests in composite materials has been widely studied. Queiroz et al. demonstrated the effectiveness of the tapping test in identifying discontinuities in polymer matrix composites, enhancing defect recognition with accelerometer and microphone signals processed through neural networks [38]. Similarly, Kim expanded this work by modeling the acoustic fields generated during tapping, illustrating its capability in identifying delamination in complex composite structures [39]. Automated advancements have further improved the consistency and precision of TT. Mohd Aris et al. highlighted the development of automated tap testers that use consistent tapping forces to map defects with higher accuracy, eliminating human subjectivity [40]. Furthermore, novel algorithms by Pools and Hartley fused force and sound data to improve clarity in identifying subsurface defects, paving the way for robotic deployment in tapping tests [41]. Recent research emphasizes the integration of TT with complementary NDT methods to overcome its inherent limitations. IRT and UT are commonly paired with TT to enhance subsurface defect detection. For example, TT can locate surface anomalies, while IRT identifies heat signatures indicative of deeper delamination or debonding [34]. This hybrid approach provides a more comprehensive evaluation of FRP-strengthened systems, improving diagnostic reliability [33].

The evolution of TT has been marked by significant technological enhancements, including automated systems, acoustic analysis tools, and AI integration. High-speed cameras with motion magnification techniques enable non-contact detection of interfacial defects, providing an efficient alternative for sensitive applications. AI-driven systems, such as those proposed by Mehrabi & Malla analyze sound patterns and classify defects with high precision, reducing operator dependency and ensuring consistent results [42]. These advancements have extended TT’s utility to large-scale infrastructure inspections, including bridges, tunnels, and retaining walls. For instance, drones equipped with automated tapping mechanisms and acoustic sensors have facilitated the inspection of inaccessible or hazardous areas, further demonstrating the method’s versatility [27]. Future research aims to refine TT by integrating it with robotics, advanced signal processing, and real-time data visualization. The development of AI-driven algorithms and digital imaging tools is expected to enhance its sensitivity and diagnostic depth, addressing existing gaps in subsurface defect detection. Additionally, hybrid NDT methods combining TT with IR, UT, and other advanced techniques will likely play a central role in monitoring FRP-strengthened concrete structures, ensuring their safety and longevity [33].

### 3.3. Impact–Echo (IE)

The Impact–Echo (IE) method has emerged as a critical NDT technique for evaluating the integrity of FRP-strengthened concrete structures. This technique leverages stress waves generated by an impact to detect internal anomalies such as delaminations, voids, and bond failures at the FRP–concrete interface. Given its effectiveness in defect detection and its cost-efficiency, IE has been extensively studied and applied in the assessment of retrofitted infrastructure. The fundamental resonant frequency (f) is related to the mechanical P-wave velocity ($\nu_{m}$) and the thickness or distance to the defect (d) of the concrete element according to the following relationship [43]:

(1) $$f=\frac{\beta \nu_{m}}{2d}$$

where β is a correction factor approximately equal to 0.96 for plate-like structures. Variations in the measured frequency indicate changes in thickness or the presence of internal defects. This correlation between wave behavior and structural anomalies underpins the diagnostic capability of the IE method.

Ekenel and Myers demonstrated IE’s capability in identifying near-surface defects, particularly delaminations caused by improper installation or environmental degradation. The findings underscore IE’s sensitivity to bond failures in FRP-strengthened reinforced concrete (RC) structures, with practical implications for bridge and building applications [44]. Pechlivani et al. proposed a 3D-printed IE device for cost-effective and practical crack detection in concrete. Their device demonstrated effective stress wave propagation detection capabilities, providing a user-friendly and accessible alternative for IE applications in infrastructure maintenance [45]. Similarly, Yu reviewed various NDT methods, highlighting IE’s effectiveness in identifying subsurface delaminations and voids in FRP–concrete systems, emphasizing its role in avoiding premature failure due to undetected defects [46].

Noël et al. validated a wireless IE prototype for concrete slab assessments, emphasizing its ability to detect voids, delamination, and cracks. The study also compared IE’s effectiveness with other NDT methods, such as GPR, and highlighted IE’s superior defect detection in the presence of reinforcement [47]. Jaishankar et al. evaluated the efficiency of IE and other advanced techniques in identifying debonding in FRP-strengthened beams, showcasing its reliability in thickness evaluation and subsurface integrity assessments [48]. Coleman et al. focused on IE’s application in RC bridge decks, demonstrating its effectiveness in locating and characterizing defects in structural components without overlays. This study reinforced the relevance of IE in large-scale structural evaluations [49]. In summary, IE has proven to be a valuable tool for evaluating FRP-strengthened concrete structures, offering a balance between effectiveness and cost-efficiency. Its ability to detect subsurface anomalies, combined with ongoing advancements in technology, positions it as a cornerstone in the field of nondestructive testing. Future research should focus on improving its accuracy for deep defects, integrating hybrid methods, and expanding its applications to large-scale and complex retrofitting projects.

### 3.4. Ground-Penetrating Radar (GPR)

Ground-Penetrating Radar (GPR) is a versatile and widely used NDT technique that has been extensively applied to evaluate the condition of EB-FRP concrete structures. GPR operates by transmitting electromagnetic (EM) waves into a structure and analyzing the reflected signals to detect internal anomalies, such as delaminations, voids, and moisture ingress. The wavelength (λ) of the EM wave is related to the EM wave velocity ($\nu_{e}$) and the signal frequency (f) by the following expression:

(2) $$\lambda =\frac{\nu_{e}}{f}$$

Similarly, the two-way travel time (t) is related to the depth (d) of the reflecting interface by:

(3) $$d=\frac{\nu_{e}t}{2}$$

The $\nu_{e}$ itself depends on the relative permittivity ($\epsilon_{r})$ of the material and is given by:

(4) $$\nu_{e}=\frac{c}{\sqrt{\epsilon_{r}}}$$

where c  is the velocity of EM in a vacuum.

**Figure 6:**
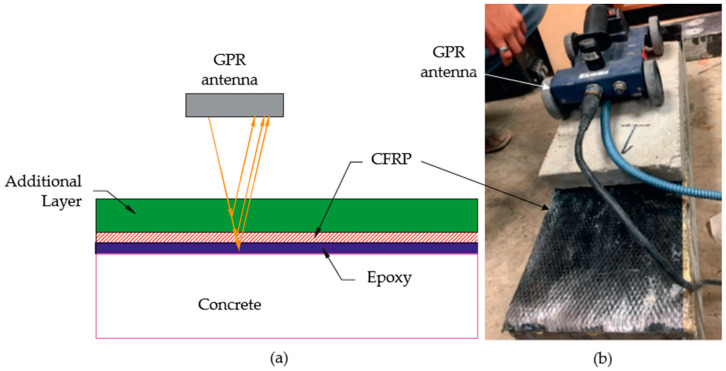
Ground-Penetrating Radar (GPR) principle of work; (**a**) GPR scanning setup, (**b**) laboratory setup with GPR antenna scanning over the EB-CFRP specimen All images adopted from [51].

Changes in reflection amplitude, time delays, and signal distortions are analyzed to identify internal anomalies. A schematic of EM wave transmission, reflection, and defect detection using GPR is illustrated in Figure 6. This technique is particularly advantageous for FRP-strengthened systems due to its ability to penetrate both the concrete substrate and the FRP layer without causing damage. Recent advancements in GPR technology, such as the development of high-frequency antennas and signal processing algorithms, have enhanced its resolution and depth of penetration, making it suitable for the evaluation of thin FRP laminates and their bond integrity with concrete [50]. However, the application of GPR for detecting defects in EB-FRP systems, particularly those utilizing carbon FRP (CFRP), is critically examined in the study by Ortiz et al. [11]. GPR is traditionally effective in identifying subsurface anomalies in concrete; however, its performance in CFRP-strengthened structures faces significant challenges. CFRP’s high electrical conductivity creates a reflective surface that severely attenuates radar signals, leading to substantial interference and masking of subsurface features. Experimental results from the study demonstrate that GPR was unable to detect critical defects such as debonding or delamination beneath CFRP layers. Furthermore, the radar’s hyperbolic reflections from the conductive surface obscured internal targets, such as steel reinforcement, making them indiscernible in post-strengthening scans [11]. Despite these limitations, the study suggests that GPR could still be effective with non-conductive FRP systems, such as GFRP (GFRP), where signal attenuation is less pronounced. Similarly, Yazdani et al. experimentally validated these limitations, noting that GPR was able to detect larger or shallow voids beneath CFRP but struggled with smaller or deeper defects [51]. In addition, the study highlighted that the need to place an additional concrete board over the CFRP surface to enhance radar signal reflection can be logistically challenging, particularly for upward-facing or overhead installations. Combined with the relatively high cost of GPR systems compared to thermal imaging equipment, these factors may limit its field practicality despite its diagnostic potential [51].

One of the most critical performance variables in GPR is antenna frequency. Higher frequencies (e.g., 1600–2600 MHz) offer greater resolution but are more susceptible to signal attenuation, which limits their depth of penetration. In contrast, lower frequencies (e.g., 800–1000 MHz) can penetrate deeper into concrete, sometimes up to 1.5 m, but at the cost of reduced resolution. This trade-off becomes vital in FRP-strengthened concrete, where signal scattering from embedded reinforcement and conductive wraps often masks internal defects. Studies have shown that 1.6 GHz antennas are effective for detecting small-scale anomalies with improved resolution [52,53], while 800 MHz systems can be advantageous for deeper inspections despite their lower detail. For instance, Almalki and Almutairi reported that an 800 MHz antenna achieved a penetration depth of 1.5 m, whereas a 1600 MHz system could only reach 0.25 m in similar RC [54]. Quinn et al. demonstrated that the use of a 2000 MHz antenna enabled precise detection of delaminations in 100–120 mm-thick polyester–glass composites, particularly under wet conditions, validating GPR’s role in marine FRP applications where dielectric contrast is enhanced [55]. To aid in selection of the appropriate GPR configuration, Table 1 summarizes typical performance characteristics of common antenna frequency ranges.

Signal clarity is influenced by the internal structure of the material. The orientation of steel reinforcement, moisture content, and the nature of the FRP layer all affect how radar signals reflect and scatter. Research by Trela et al. demonstrated that orienting antennas perpendicular to rebar improved the imaging of weak scatterers such as air voids [56]. Additionally, emerging technologies such as ultra-wideband metasurfaces have shown promise in improving signal quality and horizontal resolution at high frequencies [57]. To overcome the inherent resolution–penetration trade-off, researchers have introduced multi-frequency data fusion techniques, which combine shallow high-resolution data with deep low-resolution scans. These algorithms, based on energy balancing and frequency-domain transformations, have shown improved performance in complex FRP-reinforced geometries [58]. Additionally, novel antenna designs, such as loop bowtie and horn antennas, have enhanced detection sensitivity and reduced background clutter in near-surface inspections [59,60].

Recent advancements in signal processing and ML have significantly improved the interpretability and diagnostic performance of GPR in EB-FRP concrete structures. Traditional signal processing approaches, including wavelet denoising, independent component analysis, and time-frequency decomposition, have enhanced defect detection by improving signal-to-noise ratio and isolating features associated with bonding anomalies [61,62]. More recently, deep learning architectures, such as convolutional neural networks (CNNs) and recurrent neural networks (RNNs), have enabled the automatic classification of GPR signals, outperforming conventional methods in identifying defects such as debonding and voids [63,64,65,66,67,68]. These models are particularly effective in handling large datasets and detecting patterns in complex, noisy environments. Moreover, hybrid approaches, including transfer learning, multi-sensor fusion, and generative adversarial networks (GANs), have facilitated the integration of GPR with other NDT techniques (e.g., ITR and AE), yielding improved defect resolution in EB-FRP systems [69,70]. These innovations represent a significant step toward real-time, automated inspection platforms and are anticipated to play a critical role in advancing the reliability of GPR-based monitoring for bonded FRP applications.

When compared to other NDT methods, GPR’s limitations are stark. IRT proved more capable of identifying near-surface voids and delaminations, while PAUT successfully penetrated the CFRP layers to detect subsurface defects like concrete delamination. However, even PAUT exhibited challenges in accurately sizing certain defects. The study concludes that GPR is not a reliable standalone method for inspecting CFRP-strengthened concrete due to its inability to detect critical anomalies effectively. For such applications, methods like PAUT and IRT are recommended, as they offer greater reliability in identifying bonding and substrate defects.

### 3.5. Ultrasonic Testing (UT)

Ultrasonic Testing (UT) is a well-established NDT technique for assessing structural integrity, particularly in FRP-retrofitted concrete systems. UT operates by transmitting high-frequency sound waves into the material, detecting internal anomalies such as voids, delaminations, FRP–concrete interface, and cracks through variations in signal reflection and transmission. The wave propagates through the medium and reflects back from interfaces such as cracks, voids, delaminations, or material boundaries. The location of a defect is determined based on the time-of-flight (ToF) of the reflected pulse, governed by the following equation:

(5) $$t=\frac{2d}{\nu_{u}}$$

where $\nu_{u}$ is the ultrasonic wave velocity.

**Figure 7:**
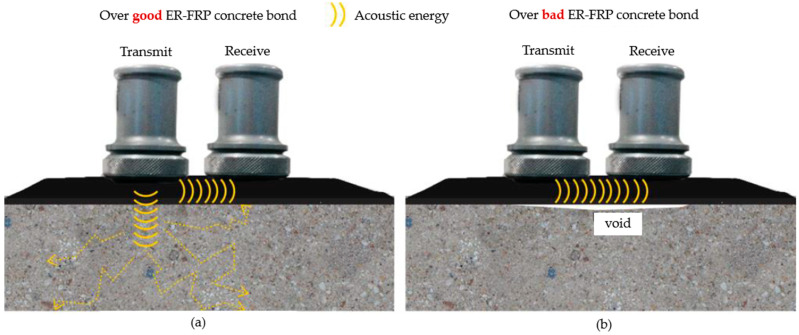
Ultrasonic wave behavior over ER-FRP concrete bonds; (**a**) over a good bond, and (**b**) over a bad bond with a void. Yellow arrows indicate the propagation path of acoustic energy used for defect detection. All images adopted from [71].

Reflections with reduced amplitude or increased travel time may indicate energy scattering, absorption, or impedance mismatch due to the presence of defects. Signal attenuation and waveform distortion are common in anisotropic or porous media such as concrete, particularly when evaluating CFRP systems where high acoustic attenuation occurs. A schematic of the pulse–echo principle for UT in EB-FRP systems is presented in Figure 7.

Recent studies have highlighted significant advancements and applications of UT in FRP systems, addressing challenges and broadening its scope. Ribolla et al. [71] presented a study focused on assessing bonding quality in FRP–concrete systems using ultrasonic techniques. This research introduced a statistical parameter called Equivalent Time Length (ETL) to quantify acoustic energy and determine bonding quality. Coupled with the Akaike Information Criterion (AIC) for automatic signal onset detection, the method demonstrated its ability to identify bonding defects in both numerical and experimental setups. Despite challenges like wave scattering in concrete, the method provided a practical approach for detecting bonding defects effectively, laying the groundwork for further advancements in UT for NDT applications [71]. Building on this work, a study by the same group further refined ultrasonic inspection techniques for detecting debonding in CFRP-strengthened concrete. The researchers enhanced the application of ETL by validating it through extensive finite element simulations and experimental trials. This study highlighted the robustness and cost-effectiveness of the approach for in-situ testing, addressing critical issues such as signal coupling inconsistencies and wave scattering. The use of ETL, unaffected by probe coupling conditions, marked a significant improvement in the reliability of ultrasonic testing for FRP–concrete systems. By quantifying acoustic energy propagation and effectively characterizing bonding conditions, this research represented a substantial step forward in SHM for FRP-strengthened concrete structures [72]. Wang et al. reviewed advancements in UT for CFRP composites, highlighting innovations in automated and intelligent NDT systems that enhance defect characterization and localization [73]. Despite its strengths, UT faces challenges with materials like CFRP due to high acoustic attenuation and anisotropy, which can obscure defect signals [74]. Advanced techniques, such as air-coupled ultrasonic testing, offer solutions by eliminating the need for coupling media, as noted by Fang et al. [75]. Additionally, Siddiqui et al. developed an empirical relationship between ultrasonic attenuation coefficients (UAC) and mechanical properties, providing insights into material quality and load-bearing capacity.

### 3.6. Phased Array Ultrasonic Testing (PAUT)

Phased Array Ultrasonic Testing (PAUT) is a powerful NDT technique utilized for inspecting FRP-retrofitted concrete structures. PAUT technology employs multiple transducer elements within a probe that can be electronically steered and focused to emit ultrasonic waves at varying angles and focal depths. This allows for enhanced detection capabilities compared to conventional UT methods. The steering and focusing capabilities of PAUT are based on constructive interference governed by the delay law [76]:

(6) $$\Delta t_{i}=\frac{x_{i}\sin\theta }{\nu_{u}}$$

where $\Delta t_{i}$ is the delay for element i, $x_{i}$ is its distance from the probe center, and θ is the beam steering angle.

One of the primary advantages of PAUT is its ability to provide high-resolution imaging with better penetration depth compared to conventional ultrasonic testing. The method allows for both line scans and area scans, producing cross-sectional and depth slice views that offer a comprehensive analysis of the internal structure [77]. In addition, PAUT in evaluating EB-FRP systems has its ability to penetrate conductive FRP layers, such as CFRP. Studies have shown that PAUT can effectively identify internal features within concrete, including delamination and embedded steel rebars, which are often challenging to detect using other methods like GPR. For instance, PAUT has been successful in detecting concrete delamination of sizes approximately 230 mm × 76 mm and rebars beneath CFRP layers [11]. However, PAUT also has limitations. While it can detect debonding defects between the first layer of CFRP and the concrete substrate, it struggles to precisely quantify the size of these defects. Additionally, PAUT’s effectiveness in detecting voids and cracks within the concrete is limited, and it may not be able to provide clear imaging of defects located immediately beneath the surface of EB-FRP systems [11]. These limitations are driven by several technical parameters. For example, amplitude attenuation in multi-layer FRP–concrete systems can reduce the signal-to-noise ratio (SNR), obscuring smaller or shallow defects [78]. Moreover, the phased array configuration, including probe frequency and aperture size, directly affects lateral resolution and focal depth, which are crucial for accurate defect dimensioning [79]. Additionally, material anisotropy in composites and complex geometries in real-world retrofits can distort wave propagation paths and reflection signatures, complicating defect localization and measurement [11]. Advances in signal processing, including ETL analysis and AIC-based signal detection, have improved PAUT’s ability to characterize flaw dimensions more reliably [71]. Furthermore, deep learning models have achieved depth prediction correlations as high as 0.95, providing promise for automated defect quantification [80]. Nonetheless, due to these inherent complexities, PAUT is most effective when used as part of a hybrid NDT strategy, complemented by techniques such as IRT or AE, to ensure robust defect assessment in EB-FRP structures.

A comprehensive review by Dolati et al. discusses the application of NDT techniques, including PAUT, for inspecting in-service FRP-strengthened concrete bridge elements. The study emphasizes the importance of selecting appropriate NDT methods based on the specific characteristics of the FRP materials and the types of defects encountered [81]. Another investigation by Mehrabi et al. evaluates the effectiveness of GPR and PAUT in detecting damage within FRP-strengthened concrete elements. The findings suggest that while GPR is effective in identifying moisture ingress and delamination, PAUT provides superior resolution in detecting internal defects such as voids and debonding at the FRP–concrete interface [42]. Furthermore, a comprehensive review by Qiwen Qiu provided an overview of imaging techniques, including PAUT, used for defect detection in FRP-bonded civil engineering structures, underscoring the importance of selecting appropriate NDT methods based on specific application requirements [82].

### 3.7. Infrared Thermography (IRT)

**Figure 8:**
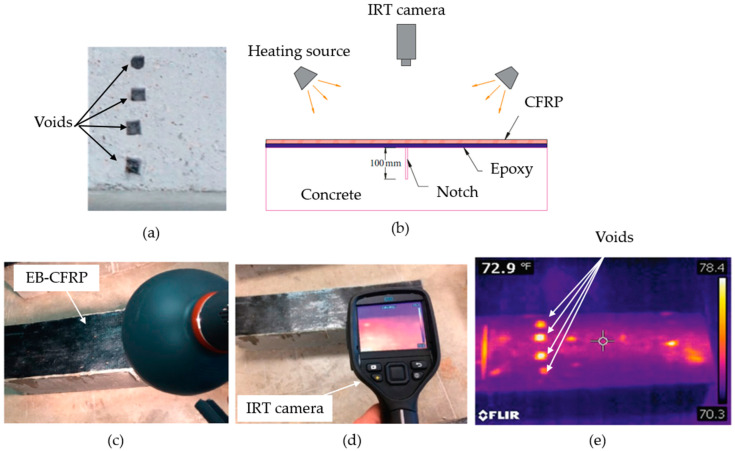
Setup and detection of artificially introduced voids in EB-FRP-strengthened concrete beams using IRT: (**a**) artificially embedded voids in concrete surface, (**b**) schematic diagram of IRT setup showing heating source, CFRP layer, and detection configuration, (**c**) surface heating, (**d**) capturing thermograph image, (**e**) thermograph of sample. All images adopted from [51].

Infrared Thermography (IRT) testing has emerged as a prominent NDT technique for assessing the condition of FRP-retrofitted concrete structures. Its widespread adoption is attributed to its capabilities in real-time inspection, non-contact measurement, and the detection of subsurface defects such as delamination and debonding. The fundamental physical principle behind IRT is heat transfer through conduction. When a structural element is subjected to external or internal heating, regions with uniform material properties exhibit uniform heat diffusion, while defects such as voids, delaminations, or debonded interfaces disrupt the thermal flow, creating localized temperature anomalies that can be detected at the surface. A schematic overview of an active IRT inspection setup for EB-FRP concrete beams is illustrated in Figure 8, showing the heating source, FRP–concrete system, and infrared camera arrangement. The heat diffusion process in solids is governed by Fourier’s law of heat conduction, and the transient thermal behavior follows the heat diffusion equation [76]:

(7) $$\frac{\partial T}{\partial t}=\alpha \nabla^{2}T$$

(8) $$\alpha =\frac{\kappa }{\rho C_{p}}$$

where T is temperature, t is time, ∇ is the Laplacian operator, and α is the thermal diffusivity, with κ representing thermal conductivity, ρ the material density, and $C_{p}$ the specific heat capacity.

Subsurface defects, typically filled with air or low-conductivity material, present much lower thermal diffusivity than the surrounding concrete or FRP. Consequently, when subjected to heating, these defective regions show delayed or accelerated thermal responses compared to sound regions, depending on the inspection strategy (heating or cooling phase). These differences are captured using infrared cameras and analyzed to identify internal defects. IRT can be performed in passive or active modes. In passive thermography, naturally occurring heat sources (e.g., solar heating) induce thermal gradients, while active thermography involves the deliberate application of an external energy source, such as flash lamps or hot air, to enhance the visibility of defects. Active IRT is often preferred for FRP–concrete structures due to its controlled heating and enhanced contrast. This section reviews recent advancements and applications of IRT in the context of FRP-retrofitted concrete structures. One of the primary applications of IRT is in quality assurance during the installation phase of FRP systems. It provides an effective means to verify the proper bonding of FRP composites to concrete substrates, identifying initial defects such as air pockets and weak bonds that could compromise the long-term performance of the strengthening system [34]. Studies have demonstrated the effectiveness of IRT in detecting common defects such as interlaminar debonding within the FRP system and at the composite–concrete interface. By analyzing thermal variations across the bonded interface, IRT facilitates the mapping of damage extent and the early identification of potential failure zones [34]. Yazdani et al. conducted a comprehensive experimental investigation on small-scale concrete beams strengthened with CFRP laminates under various controlled defect conditions, including embedded voids, surface wetness, and contamination. Their results showed that IRT effectively detected and localized subsurface debonding areas by highlighting distinct thermal anomalies. Quantitative analysis revealed that thermographic evaluations slightly overestimated the debonded areas by approximately 24% compared to the actual defect sizes; however, the technique provided reliable early-stage detection of defects that could compromise long-term structural performance [51].

In addition to defect detection, IRT plays a crucial role in monitoring the progression of damage in FRP-strengthened structures under cyclic loading and environmental exposure. This long-term monitoring capability allows for the assessment of the durability and structural integrity of FRP systems, making it a valuable tool for infrastructure maintenance and rehabilitation. A study by Brown and Hamilton demonstrated the efficacy of IRT in identifying subsurface defects in FRP systems applied to concrete, utilizing single-pixel analysis to achieve quantitative assessments [83]. Further research by Mtenga et al. emphasized the importance of quality assurance in FRP retrofits, highlighting IRT’s capability to detect bonding issues that could compromise structural integrity [84]. Moreover, a comprehensive review by Yumnam et al. discussed active IRT techniques, underscoring their applicability in inspecting concrete structures reinforced with FRP composites [32].

IRT offers several advantages that make it a preferred choice for the inspection of FRP-strengthened structures. It provides rapid, real-time analysis with minimal setup requirements, allowing for efficient inspections without disrupting ongoing operations. The non-contact nature of the technique minimizes the risk of further structural damage and enables the detection of hidden defects that might not be visible through surface inspections. Studies such as Halabe et al. confirmed IRT’s effectiveness in rapidly evaluating the subsurface conditions of FRP composite bridge decks and other structural elements [85]. IRT is often used in conjunction with other traditional inspection techniques such as VT inspection and TT, offering a more comprehensive evaluation of structural conditions. This integration enhances the reliability of defect detection and provides engineers with critical insights into the performance of the FRP retrofit over time. Despite these advantages, IRT has certain limitations that must be considered. The technique relies on the presence of an adequate heat source to generate a detectable thermal gradient, which may be influenced by environmental factors such as ambient temperature and wind. Additionally, variations in surface emissivity can impact the accuracy of defect detection, and the depth of penetration is limited by the thermal conductivity of the materials involved [25].

### 3.8. Acoustic Impact Testing (AIT)

Acoustic Impact Testing (AIT) is a widely used NDT technique for assessing the condition of FRP-strengthened concrete structures. It involves striking the surface of the structure with an impact device, such as a hammer or tapper, and analyzing the resulting acoustic signals to detect defects such as delaminations, voids, and debonding at the interface between the FRP and the concrete substrate. Studies have shown that AIT is particularly effective for detecting near-surface defects in FRP-strengthened concrete structures. The technique is cost-effective, simple to implement, and provides immediate feedback on the condition of the structure. One significant application of AIT is in the detection of hidden defects in FRP systems that undergo cyclic loading. By monitoring sound wave propagation through the structure, engineers can identify changes that indicate the onset of deterioration. Studies have shown that acoustic impact testing can effectively detect and localize damage, helping to ensure the long-term structural integrity of FRP retrofits [11].

The advantages of AIT include its ability to provide detailed information about subsurface defects without causing damage to the structure. It is a relatively simple and cost-effective technique that can be conducted with minimal equipment. However, the method’s effectiveness can be influenced by external noise and environmental conditions, requiring careful implementation to achieve accurate results. Additionally, AIT has limitations, including its reliance on operator experience and subjectivity in interpreting results. The accuracy of the technique can be affected by factors such as surface roughness, environmental noise, and the presence of multiple layers of materials in the structure. Moreover, AIT is generally limited to detecting defects close to the surface and may not be as effective for identifying deep-seated anomalies. Recent advancements in AIT include the integration of digital TT, which combines traditional impact testing with digital sensors and data analysis tools. This allows for more precise defect localization and quantification, improving the reliability and repeatability of the technique.

### 3.9. Eddy Current Testing (ECT)

Eddy Current Testing (ECT) is a widely used NDT technique for assessing FRP-retrofitted concrete structures, especially those incorporating CFRP composites. The method operates by inducing eddy currents within the conductive carbon fibers using an alternating current (AC) coil. Disruptions in the current flow, caused by defects such as cracks, fiber damage, or delaminations, are detected and analyzed to identify structural anomalies [16]. CFRP materials are particularly well-suited for ECT due to their electrical conductivity, allowing for effective detection of fiber-related defects. However, the method has limitations when it comes to identifying damage related to the polymer matrix or internal voids. The performance of ECT can also be influenced by various factors, including probe lift-off distance, the orientation of defects in relation to the current flow, and the material’s conductivity properties. Additionally, the use of high-frequency excitation may be necessary to ensure effective interaction with CFRP materials, which in turn restricts the penetration depth of the inspection [25].

ECT methods have been explored as a potential solution for detecting construction defects and environmental damages in FRP-strengthened concrete structures. Yu highlights the importance of NDT techniques in assessing the integrity of FRP retrofits, emphasizing the role of ECT in evaluating steel reinforcements beneath FRP layers [46]. The study identifies variations in eddy current signals as indicators of delamination and potential structural degradation in FRP–concrete bonds. Heuer et al. [86] establish high-frequency ECT (1–50 MHz) as a superior NDT method for CFRP composites, overcoming PAUT and IRT limitations in detecting fiber misalignment, discontinuities, and resin inconsistencies. Their phase rotation technique effectively compensates for surface roughness, enhancing subsurface defect visualization. While ECT enables contactless, high-sensitivity flaw detection, its anisotropic conductivity dependence necessitates adaptive signal processing. Despite proving ECT’s viability for CFRP-strengthened structures, its integration with AI-driven analysis and multi-sensor fusion remains critical for future advancements [86]. Morozov et al. established Capacitive Imaging (CI) as a high-frequency (200 MHz) NDT method for detecting impact-induced delaminations in CFRP composites, outperforming UT and ECT in imaging shallow defects. CI leverages dielectric variations in the resin matrix, enabling subsurface damage detection where ECT fails due to anisotropic conductivity [87]. However, Du et al. developed a fast forward solver and hybrid inversion scheme for ECT of anisotropic CFRP laminates, enabling accurate crack reconstruction. Their approach integrates an electric conductivity tensor for precise ECT signal simulation and a Conjugate Gradient (CG)–Genetic Algorithm (GA) hybrid for optimized defect sizing. Experimental validation confirms high-fidelity crack reconstruction, though ECT remains ineffective for delaminations and matrix cracks [88].

### 3.10. Acoustic Emission (AE)

**Figure 9:**
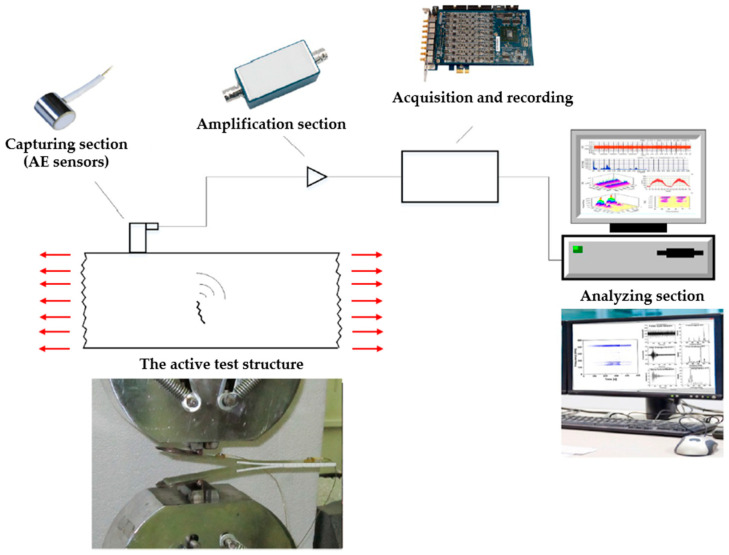
The different units of an AE monitoring system. The red arrows indicate the stress waves emitted from the structure under mechanical loading. These waves are detected by the AE sensors during the AE monitoring process [89].

Acoustic Emission (AE) is a passive NDT technique that detects transient elastic waves generated by the rapid release of energy within materials. This method is particularly useful for monitoring crack propagation, fiber fractures, matrix microcracking, fiber-matrix debonding, and delamination in composite structures such as FRP composites [10]. Unlike conventional UT, AE does not require an external energy source but instead relies on capturing naturally occurring stress waves within a material under load [12]. The method is highly sensitive to defect initiation and growth, making it an excellent tool for real-time monitoring of structural integrity. The AE process begins with an AE sensor network placed on the surface of a structure to capture minor vibrations as the material undergoes stress. The detected signals are then amplified, digitized, and processed to extract relevant characteristics such as amplitude, energy, duration, hit count, and rise time [13,89]. A key feature of AE analysis is source localization, which helps identify the origin of damage. Various signal processing techniques, including Fast Fourier Transform (FFT) and ML algorithms like Principal Component Analysis (PCA), are used to classify and interpret AE signals [90]. The equipment required for AE monitoring is explained in Figure 9.

AE has been widely applied in several domains, particularly in monitoring damage progression in FRP composites and concrete structures. For instance, Choi and Yun investigated the AE activity of CFRP-strengthened RC beams exposed to freeze–thaw cycles. Their study demonstrated that AE parameters such as event counts, energy, amplitude, and frequency effectively reflected damage progression. Notably, AE activity decreased significantly after prolonged freeze–thaw cycling, indicating material degradation. The results suggested that AE monitoring is a reliable tool for assessing the long-term durability of CFRP-strengthening systems [91]. Nair et al. demonstrated AE’s effectiveness in tracking failure progression in CFRP-strengthened concrete beams but noted challenges in distinguishing failure modes due to signal complexity, advocating for spectral analysis and pattern recognition [92]. Their study advanced this by integrating k-means clustering and neural networks, enabling automated classification of AE signals into distinct damage mechanisms such as matrix cracking, microcracking, and debonding [93]. The evolution from signal-based monitoring to AI-enhanced classification underscores AE’s growing role as a precise, real-time tool for SHM in CFRP-strengthened concrete.

Ghiassi et al. applied AE techniques to characterize debonding failure in FRP-strengthened masonry structures. Their study found a clear correlation between AE signal parameters and different debonding mechanisms, making AE an effective tool for monitoring bond integrity. The results showed that cumulative AE energy was closely linked to FRP slip and debonding fracture energy, providing a quantitative approach for evaluating structural performance [94]. Holsamudrkar and Banerjee [95] explored AE-based SHM of full-scale RC beams strengthened with fiber-reinforced cementitious matrix (FRCM) composites. Their study demonstrated that AE cumulative hits, signal strength, and historical index could effectively distinguish different failure phases. The AE monitoring technique was particularly useful in identifying critical damage points and assessing the effectiveness of mechanical anchorage in improving load capacity [95]. Ma and Li investigated the effectiveness of AE for assessing crack states in FRP-strengthened RC columns subjected to cyclic loading. They found that AE techniques could effectively track crack initiation and propagation, enabling a four-stage classification of structural damage. Their study also introduced a fractal-based AE damage index, which correlated well with energy dissipation and provided a novel approach for real-time SHM [96].

### 3.11. Radiographic Testing (RT)

Radiographic Testing (RT), including both X-ray and gamma radiography, is an NDT technique that inspects internal features of materials by transmitting high-energy photons through a structural element and recording the attenuated radiation on a detector or photographic film positioned on the opposite side [76]. In FRP-strengthened concrete structures, RT can be applied to detect internal anomalies such as voids, cracks, delaminations, and other subsurface defects that are not observable by surface inspection. By exploiting differences in material density and thickness, RT provides valuable imaging of internal discontinuities, although its effectiveness is influenced by factors such as material composition, member thickness, and the contrast between the FRP layers and the concrete substrate. The fundamental physical principle underlying RT is the exponential attenuation of radiation as it passes through matter, governed by the Beer–Lambert law [76]:

(9) $$I=I_{0}e^{-\mu x}$$

where I is the transmitted radiation intensity, $I_{0}$ is the initial incident intensity, μ is the linear attenuation coefficient of the material (dependent on material density and atomic number), and x is the material thickness along the radiation path.

Despite its effectiveness in industrial sectors such as aerospace and manufacturing, the application of RT for inspecting EB-FRP concrete structures is extremely limited. Several practical challenges restrict its viability. First, the high density and substantial thickness of concrete members result in significant attenuation of radiation, necessitating the use of extremely high-energy sources, which are impractical and pose safety risks for routine field inspections [13,16,27]. Second, the density contrast between FRP laminates and the underlying concrete substrate is relatively small, thereby reducing the visibility of defects such as debonding or thin delamination zones. Third, RT generally requires access to both sides of the inspected element for positioning the radiation source and detector, a condition that is rarely achievable in large civil infrastructure such as bridge decks, columns, or slabs [13,25,97]. Moreover, the strict regulatory and safety requirements associated with the use of ionizing radiation further complicate the deployment of RT systems in open field environments [14,27,98,99,100].

Although no field-scale applications of RT for EB-FRP-strengthened concrete structures have been reported, recent laboratory-based studies employing high-resolution X-ray micro-computed tomography (Micro-CT) have contributed to a deeper understanding of interfacial behavior. Riccardi et al. utilized in-situ X-ray Micro-CT imaging, coupled with Digital Volume Correlation (DVC) techniques, to investigate the debonding mechanisms of CFRP anchors embedded in small-scale concrete beams [101]. Their approach enabled the real-time tracking of material degradation and the evolution of internal cracking under mechanical loading. Microtomography provided detailed three-dimensional insights into the progression of pull-out failure and damage accumulation at the FRP–concrete interface. However, despite the success of Micro-CT in capturing these phenomena at the laboratory scale, the practical application of conventional RT or CT methods for large-scale civil infrastructure remains severely limited due to constraints related to concrete thickness, radiation attenuation, equipment portability, and safety regulations. As such, while Micro-CT serves as a valuable experimental tool for fundamental studies, nondestructive field evaluation of EB-FRP concrete structures continues to rely predominantly on UT, IRT, AE, and GPR methods.

## 4. Discussion

### 4.1. Comparative Evaluation of NDT Methods

The NDT methods reviewed in this study demonstrate significant potential in assessing EB-FRP systems used in concrete retrofitting. However, the effectiveness, limitations, and practicality of these techniques vary, necessitating a comparative evaluation to determine their applicability in real-world scenarios. A summary of the strengths and limitations of the reviewed NDT methods is presented in Table 2. Each method is evaluated based on its capability to detect common defects in EB-FRP systems, such as debonding, delamination, voids, and cracks, along with its practical feasibility in large-scale infrastructure assessments. The comparison highlights that no single NDT method is universally superior; instead, their combined application provides more reliable structural assessments. Methods like PAUT and UT offer higher depth penetration but require skilled operators, while IRT and GPR are effective for surface-level evaluations but have limitations based on material properties.

The cost–performance ratio of NDT methods is a critical factor in their practical adoption. VT and TT methods are cost-effective but lack reliability in detecting subsurface defects. UT and PAUT offer improved defect characterization at a moderate cost, though they require skilled operators. RT, while providing the most detailed internal assessments, is the most expensive method due to equipment and safety requirements, making it impractical for routine inspections. GPR and IRT balance cost and efficiency, offering mid-range affordability with reasonable detection capabilities. A hybrid approach that combines low-cost preliminary screening methods (e.g., VT and TT) with targeted high-accuracy techniques (e.g., PAUT, AE) could optimize cost and performance, enabling widespread field applications.

### 4.2. Comparative Performance for AI/ML Algorithms in NDT of EB-FRP Structures

The successful application of AI and ML in NDT of EB-FRP concrete structures requires a careful selection of algorithms that align with the nature of sensor data, the type of defects to be detected, and operational deployment constraints. Across various NDT methods, ranging from image-based (e.g., IRT) to time-series-based (e.g., AE, UT), ML models have demonstrated significantly improved performance in classification, defect quantification, and anomaly detection. For image-based NDT techniques, convolutional neural networks (CNNs) and their variants (e.g., Mask R-CNN, U-Net) have shown superior defect segmentation and localization accuracy, with F1-scores often exceeding 95% in well-labeled datasets [98]. In time-series or waveform-based NDT, models like recurrent neural networks (RNNs), 1D CNNs, and Support Vector Machines (SVMs) are commonly used. For example, Zhang et al. demonstrated that 1D CNNs trained on AE signals achieved over 97% accuracy in identifying defect classes in composite concrete systems. Also, a study reported classification accuracies exceeding 90% when SVM was applied to IE signals for distinguishing corrosion-induced defects in FRP-reinforced concrete elements [102]. Similarly, ANN-SVM hybrids have been employed in interpreting IE frequency shifts, offering practical accuracy above 90% in differentiating delamination from voids [98]. In more complex settings, ensemble models (e.g., Random Forest (RF), Gradient Boosting, CatBoost) and hybrid deep learning frameworks (e.g., CNN + long short-term memory (LSTM)) are increasingly adopted to combine decision strengths and improve robustness. Recent advances also show that transfer learning and data fusion across modalities (e.g., combining IRT and visual data in CNNs) improve generalization and fault isolation accuracy, especially when data from a single method are ambiguous. Interpretability remains essential in engineering practice. Models like decision trees and SVMs are favored when transparency is critical, while deep models are increasingly acceptable if visual outputs or saliency maps are used for explanation. Real-time deployment is feasible for lightweight CNNs and pruned architectures running on embedded systems, particularly for GPR, IRT, and AE monitoring platforms. Table 3 summarizes the recommended AI/ML models for major NDT methods, their typical performance, and key deployment considerations in EB-FRP applications.

### 4.3. Research Gaps and Standardization Challenges

Despite significant progress in NDT technologies for the assessment of FRP-strengthened concrete structures, critical research gaps and standardization challenges persist. These issues limit the effective and widespread adoption of advanced NDT methodologies in large-scale infrastructure monitoring. The following key challenges are identified:

- Lack of unified testing standards: currently, there are no universally recognized protocols governing the use of NDT for EB-FRP systems. This absence has led to inconsistencies in defect detection, data interpretation, and overall evaluation across different studies and field practices. Recent frameworks, such as [24], have emphasized that standardized inspection approaches for concrete systems reinforced or strengthened with FRP remain under active development, requiring further harmonization efforts.
- Limited integration of AI and ML: although AI and ML techniques have demonstrated promising capabilities in automating defect detection and classification, their application in FRP inspection remains at an early experimental phase. Challenges include limited availability of large, labeled datasets; difficulty in generalizing models across different FRP configurations; and the need for domain-specific AI validation protocols to ensure reliability under diverse field conditions.
- Insufficient validation of hybrid inspection frameworks: hybrid NDT frameworks, which combine complementary techniques such as GPR with PAUT or IRT with AE, offer theoretical advantages in defect detection and characterization. However, comprehensive experimental validations and comparative performance evaluations for these hybrid schemes are still lacking. As highlighted by a recent report [24], practical implementation of hybrid inspection workflows necessitates further development of computational models, field validation campaigns, and standardized interpretation protocols.
- Economic feasibility and scalability issues: the high cost associated with advanced NDT methods, particularly PAUT and RT, poses barriers to routine implementation in infrastructure asset management. Future research should prioritize the development of cost-optimized hybrid approaches that balance diagnostic performance with operational affordability, enabling broader adoption across different project scales and resource settings.
- Challenges in real-time remote sensing deployment: the integration of unmanned aerial vehicles (UAVs) equipped with advanced NDT sensors (e.g., IRT cameras, wireless AE systems) holds significant promise for large-area, rapid assessments of FRP-retrofitted structures. Nevertheless, technological barriers related to real-time data acquisition, transmission, processing, and autonomous defect localization must be overcome. Further field trials and protocol development are necessary to enable fully automated, high-confidence remote inspections.

In summary, while hybrid and AI-enhanced NDT approaches present exciting opportunities, their maturity levels vary widely across applications. Systematic efforts in standardization, rigorous field validations, and interdisciplinary collaborations between material scientists, data scientists, and structural engineers will be pivotal to fully unlocking the potential of NDT for FRP infrastructure assessment.

### 4.4. Future Directions

Recent advances in NDT technologies have introduced ML and AI-enhanced defect detection, automated robotic inspections, and novel sensing technologies for improved structural health monitoring [109]. The growing use of deep learning models for automated crack detection, AI-assisted IE signal processing, and hybrid AI-UT analysis have demonstrated promising improvements in detection accuracy. Recent studies have highlighted the need for continued development in this field, particularly in refining training datasets and improving model generalizability. In addition to AI-based approaches, there are emerging NDT techniques such as laser-based ultrasonic testing (LUT), which employs laser-generated ultrasonic waves for high-resolution defect detection in FRP structures. It provides non-contact, high-precision measurements, making it ideal for delicate composite materials. Additionally, there is digital image correlation (DIC), which is a full-field optical technique that measures strain and deformation in structures by tracking surface patterns. It is particularly useful for monitoring micro-deformations in FRP-strengthened concrete systems. Moreover, fiber-optic sensing (FOS) for continuous SHM health monitoring is gaining traction. These novel methods have demonstrated the potential in enhancing defect characterization, particularly for real-time monitoring and predictive maintenance applications. However, further experimental validation and standardization efforts are required before widespread industry adoption. Furthermore, advancements in drone-assisted NDT inspections, particularly for large-scale infrastructure such as bridges and high-rise structures, have introduced the possibility of real-time defect mapping through high-resolution IRT and UAV-based acoustic impact testing. These developments represent a significant shift toward more automated and scalable inspection techniques, reducing the reliance on labor-intensive manual assessments. To enhance the effectiveness of NDT methods in EB-FRP applications, several key areas should be prioritized in future research and development:

- Integrating AI-driven automation: expanding deep learning frameworks to enable more accurate and autonomous defect detection.
- Hybrid NDT solutions: developing and validating integrated approaches that combine multiple NDT techniques (e.g., PAUT-GPR or IR-AE hybrid systems).
- Advancing remote and UAV-based inspections: enhancing drone-assisted inspections for large-scale infrastructure assessments.
- Cost-effective NDT solutions: improving affordability through innovative sensor designs and efficient testing protocols.
- Standardization of novel techniques: establishing regulatory frameworks for emerging technologies such as LUT, FOS, and DIC in FRP structure monitoring.

## 5. Conclusions

Externally bonded FRP systems have become vital in extending the service life of deteriorating concrete structures, and this review has consolidated the state-of-the-art in how such systems can be inspected and safeguarded using NDT techniques. In summary, the core contribution of this review is a comprehensive evaluation of existing and emerging NDT methods for EB-FRP concrete: it catalogues the capabilities of traditional approaches (visual inspection, tapping, impact–echo, ultrasonic, and electromagnetic methods, etc.) and situates them within modern SHM frameworks that increasingly exploit automation and AI. The review makes it clear that while each NDT method offers unique insights—for example, GPR can peer into concrete depths and PAUT can image internal features with high resolution—no single technique provides a holistic assessment of an EB-FRP system’s integrity. This underscores one key finding: hybrid approaches that combine multiple techniques are essential to capture the multi-faceted nature of FRP debonding, delamination, and other defects. By comparing methods side-by-side (in terms of defect detectability, penetration depth, sensitivity, and practical constraints), the paper provides practitioners and researchers with guidance on optimal inspection strategies for various scenarios.

Despite the technological advancements documented, this review also highlights several major challenges that the field must address. First, standardization is lacking, there are currently no universally adopted protocols or criteria to evaluate NDT results on EB-FRP concrete structures, leading to inconsistent interpretations across different studies and projects. Developing unified testing standards and calibration procedures is critical to ensure that defect diagnoses are reliable and comparable. Second, certain physical limitations of NDT methods remain unsolved—for instance, the severe signal attenuation caused by conductive CFRP laminates in GPR, or the difficulty of ultrasonic waves in characterizing near-surface defects—and these limit the effectiveness of individual methods. Third, the integration of advanced technologies such as AI and hybrid sensor systems is still in its infancy for FRP applications; data-driven algorithms require large, high-quality datasets for training, and the FRP community has only begun to compile such data. Moreover, theoretical advantages of combining methods (e.g., GPR with PAUT, or IRT with AE) have yet to be fully validated by extensive field trials. Economic and scalability issues also pose challenges, many high-performance NDT techniques involve expensive equipment and specialized expertise, which can impede their routine use in large infrastructure networks.

Looking forward, the future of NDT for EB-FRP structures lies in overcoming these challenges through innovation and collaboration. Future research directions should prioritize the following: (1) hybrid NDT solutions, designing integrated inspection workflows and custom sensor suites that leverage the complementary strengths of different methods, and validating them on real structures to establish their reliability and added value; (2) AI-driven data analysis, developing robust ML models (including deep learning and data fusion techniques) that can automatically interpret complex NDT signals or images, distinguish defect signatures from noise, and even predict the evolution of damage, all while providing explainable outputs that engineers can trust; (3) automation and remote sensing, expanding the use of drones and robotics equipped with NDT sensors to conduct rapid, high-resolution surveys of large or difficult-to-access structures (such as bridge girders or high-rise façades), thereby reducing inspection time and human risk; (4) cost-effective monitoring strategies, exploring low-cost sensors and smart monitoring systems (for example, networks of fiber-optic strain sensors or inexpensive acoustic sensors) that can continuously track the health of FRP retrofits, alerting inspectors only when anomalies arise, which would optimize resource allocation; and (5) standardization efforts, working through professional societies and research consortia to establish standardized test methods, certification programs for NDT of FRP-strengthened structures, and benchmark case studies that can serve as references for performance. By addressing these areas, the field will move closer to ensuring that NDT techniques can fully support the safe and efficient use of FRP in infrastructure rehabilitation.

In conclusion, the evolution of NDT methodologies, enriched by hybrid techniques, informed by AI, and guided by emerging standards, is poised to significantly enhance the maintenance of FRP-strengthened concrete structures. This review serves as a foundational reference, charting what has been accomplished and what challenges lie ahead. With continued research and interdisciplinary collaboration, the innovative NDT solutions discussed here will mature into practical tools for engineers, ensuring that the impressive gains offered by FRP retrofitting are protected by equally advanced inspection and monitoring capabilities. Through such progress, stakeholders can expect safer, more resilient infrastructure that confidently leverages both the strength of composite materials and the precision of modern nondestructive evaluation.

## Figures and Tables

**Table 1:** Typical Performance trade-offs of GPR antennas for FRP-strengthened concrete.

Frequency Range (MHz)	Typical Penetration Depth	Resolution	Recommended Use
800–1000	Up to 1.5 m	Low	Deep subsurface inspections, thick concrete
1000–1600	Up to 0.40 m	Moderate	Balanced inspections in medium-depth areas
1600–2600	Up to 0.25 m	High	Near-surface defects, bond quality evaluation

**Table 2:** Comparative evaluation of NDT methods for EB-FRP systems.

NDT Method	Detects Debonding	Detects Voids	Detects Delamination	Depth of Penetration	Cost	Automation Potential	Key Limitations
VT	No	No	No	Surface only	Low	Low	Operator dependent, subjective results
TT	Yes (Limited)	No	Yes	Surface only	Low	Low	Qualitative, operator dependent
IE	Yes	Yes	Yes	Moderate (~200 mm)	Medium	Moderate	Requires expertise, affected by material heterogeneity
GPR	Limited (CFRP)	Yes	Yes	High (~500 mm)	Medium-High	High	Ineffective for conductive CFRP
UT	Yes	Yes	Yes	Moderate (~300 mm)	Medium	Moderate	Requires coupling medium, sensitive to surface roughness
PAUT	Yes	Yes	Yes	Moderate (~350 mm)	High	High	High cost, requires skilled operators
IRT	Yes	No	Yes	Shallow (~50 mm)	Medium	High	Sensitive to environmental conditions
AIT	Yes	No	Yes	Surface to moderate (~150 mm)	Medium	Moderate	Operator dependent, susceptible to environmental noise
ECT	No	No	Yes (for CFRP)	Shallow (~5 mm)	High	Moderate	Limited to conductive materials
AE	Yes	No	Yes	Deep (varies)	High	High	Requires load application, noise interference
RT	Yes, lab-scale	Yes	Yes	High (~500 mm)	Very High	Moderate	High radiation exposure, limited field use, costly

**Table 3:** Comparative summary of AI/ML models for NDT methods applied to EB-FRP concrete structures, adopted from [17,62,63,64,98,102,103,104,105,106,107,108].

NDT Method	Typical AI/ML Models	Accuracy/F1 Score	Use Case Suitability
VT	CNN, Transfer Learning	95–98%	Crack and surface flaw classification
TT	SVM, RF, ANN	70–85%	Qualitative bond check, delamination cues
IE	SVM, 1D CNN, Hybrid ANN	80–90%	Voids, debonding, frequency domain analysis
GPR	1D CNN, RNN	85–95%	Real-time damage evolution and type classification
UT	1D CNN, ANN, SVM	80–90%	Interface debonding detection
PAUT	2D CNN, SVM, RF, Transfer Learning	85–99%	Subsurface delamination and rebar localization
IRT	CNN, U-Net, Mask R-CNN	90–97%	Internal delamination and interface flaw identification
AIT	ANN, CNN, SVM	90–95%	Imaging of CFRP defects, bond evaluation
ECT	SVM, RF, CNN	90–96%	Surface/subsurface debonding detection
AE	1D CNN, RNN, SVM	85–95%	Conductive CFRP integrity analysis
RT	CNN, Transfer Learning	90–95%	Deep internal voids and FRP delamination visualization

## Data Availability

The data presented in this study are available on request from the corresponding author.
